# Effects of Community Environment, Leisure, and Social Activities on Health Status of Older Adults with Diabetes in South Korea

**DOI:** 10.3390/healthcare11142105

**Published:** 2023-07-24

**Authors:** Jiyoun Kim, Yoonho Ra, Eunsurk Yi

**Affiliations:** 1 Department of Exercise Rehabilitation, Gachon University, Incheon 21936, Republic of Korea; eve14jiyoun@gachon.ac.kr; 2 Institute of Human Convergence Health Science, Gachon University, Incheon 21936, Republic of Korea; yhra0801@gmail.com

**Keywords:** community environment, leisure activities, social activities, health status, older adults, diabetes, South Korea

## Abstract

This study investigates the effects of community environment, leisure, and social activities on the health status of older adults with diabetes, a serious disease in modern society. Data from the 2020 National Survey of Older Koreans were analyzed. Descriptive statistics were used to assess participants’ characteristics, and regression analyses were conducted to assess the effects of community environment, leisure, and social activities. Mediating effects were tested using hierarchical regression analysis and bootstrapping. The key results are as follows. (a) Community environmental satisfaction affected participation in leisure and social activities. (b) Community accessibility had a negative effect on subjective health, while community environmental satisfaction had a positive effect on subjective health, cognitive function, and chronic diseases. (c) Leisure activities had a positive effect on cognitive health, while social activities influenced subjective health, cognitive function, and chronic diseases. (d) Analysis of the mediating effect of leisure and social activities on the relationship between the community environment and health status of older adults with diabetes confirmed a partial mediating effect. To improve older adults’ mental and physical health, mere quantitative increases in the community environment will not be sufficient. It is necessary to cultivate and manage professionals to increase opportunities for participation by increasing social exchanges and systematically managing older adults’ health.

## 1. Introduction

As life expectancy and healthy life expectancy increase, self-reliance and health in older age become socially important. There is increasing interest in aging in place (AIP), which involves individuals living independently as members of society in the community, rather than in a hospital or facility, and spending life meaningfully in older age. Humans interact with their social and physical environments and create meaningful representations of themselves within the environment over time [1]. The World Health Organization referred to communities with policies and environments that support such active aging as “age-friendly cities” [2]. Various community environmental factors in these communities have a positive impact on the lives of older adults through interactions with each other [2,3].

Since the residential environment surrounding human beings is a critical factor in life, the government of the Republic of Korea recognizes residential environment as a basic human right. Article 35 of the Constitution states the following: “All citizens have the right to live in a healthy and pleasant environment...the people should strive for environmental preservation” [4]. Institutional improvements for older adults have also been discussed considering the rapidly aging population in Korea. This included the introduction of a long-term care insurance system for older adults in 2008 and an increase in the number of care facilities and hospitals targeting older adults. Policies have also been implemented to improve older adults’ residential environment, such as the Housing Affirmative Support Act in 2012, which deals with the provision of support for persons with disabilities and older adults [5].

In older age, people spend more time at home because their life is centered around their family rather than society, and their attachment to the residential environment is higher than that of other age groups [6]. Moreover, older adults tend to focus more on forming and maintaining new social relationships [7] and spend significant time interacting with neighbors [8]. Therefore, the national and local governments in Korea are building community environments for older adults considering their desire to improve their residential environment and social relationships.

As the topic of life in older age gains greater importance, older adults’ health and environmental factors must be considered. In particular, various studies have been conducted on chronic diseases (heart disease, cancer, hypertension, diabetes, etc.) with high mortality rates that directly influence the healthy life of older adults. Deterioration due to chronic diseases, such as diabetes, depression, and osteoarthritis, causes mobility problems in older adults and reduces voluntary physical activity [9]. In particular, diabetes has become a global issue due to a rapid increase in mortality and complications, and patients diagnosed with diabetes now also include young adults and adolescents. The increasing proportion of persons with diabetes is becoming a broader social problem [10]. Diabetes is affected by lifestyle, and lifestyle is affected by the environment. A good environment can change one’s lifestyle, and a good lifestyle can be expected to decrease the prevalence of diabetes or prevent the disease altogether [11,12,13].

The Republic of Korea became an aging society in 2000 and an aged society in 2017. As of May 2023, the older adult population accounted for 18.4% (9,499,933) of the total population (51,558,034), making Korea the most rapidly aging country in the world [14]. The annual average rate of patients with a confirmed diagnosis of diabetes is 5.6%, and middle-aged and older adults account for the largest percentage of confirmed patients [15]. In addition, due to the growth of delivery services, individuals’ activity levels have been reduced and eating habits have become problematic, which have exposed all ages to the risk of diabetes. In Korea, where the demographic structure is an inverted pyramid, it is necessary to examine the relationship between the community environment, activities of older adults, and their health to prepare more realistic health measures for older age.

Therefore, this study aims to analyze the relationship between the community environment, leisure, and social activities and health trends of older adults diagnosed with diabetes using data from the National Survey of Older Koreans, which is conducted for people aged 65 years and above by the Korea Institute for Health and Social Affairs. These results are expected to be used as a basis for developing measures related to community and external activities for improving the health of older adults diagnosed with diabetes in Korea.

## 2. Materials and Methods

### 2.1. Data

This study used the original data from the 2020 National Survey of Older Koreans conducted by the Korea Institute for Health and Social Affairs. This survey has been conducted every three years since 2008 as a statutory survey based on Article 5 of the Elderly Welfare Act. The survey was conducted with 10,097 older people aged 65 years or older living in Korea. The 2020 survey included 10 items: general households, health status and health behavior, functional status and care, leisure and social activities, economic activities, family and social relations, exchange of family help, living environment and older age, cognitive function, and economic condition. This study was conducted in accordance with the guidelines of the Declaration of Helsinki and approved by the Institutional Review Board of the Korea Institute for Health and Social Affairs (No. 2020-36, 8 July 2020). This study excluded the data of participants who did not respond themselves and the data of subjects who were not diagnosed with diabetes. Finally, the participants responded to the questionnaire directly, and the analysis used data from 2376 people diagnosed with diabetes. The survey data were analyzed based on health status, health behavior, leisure and social activities, living environment, and response data.

### 2.2. Variables

Table 1 presents the variables of this study. The independent variable measured accessibility and satisfaction regarding the community environment. Accessibility was measured by the following question: “How long does it take to get to the following institutions from the house you live in?” The items included (1) places to purchase daily products, such as markets and supermarkets; (2) hospitals and health centers; (3) administrative welfare centers; (4) social welfare centers and women’s centers; (5) bus stops and subways; and (6) parks for walking and exercises. The response options ranged from 1 (less than 5 min on foot) to 5 (more than 30 min on foot). Satisfaction was assessed using the following question: “How satisfied are you with the community environment you currently live in?” The items included (1) streets of convenience facilities, social welfare facilities, and medical institutions; (2) public transportation frequency/route; (3) green space sufficiency/distance; (4) security and traffic safety; (5) distance from the residence of a child or relative; (6) opportunities to interact with neighbors; and (7) overall community environment. The response options ranged from 1 (very satisfied) to 5 (not satisfied at all).

The mediating variable measured participation in leisure and social activities. Leisure activities included watching TV, listening to the radio, traveling, leisure culture, and educational activities. The following yes/no questions were used to assess participation in leisure activities: “Have you ever watched TV or listened to radio in the past year?”; “Have you traveled in the past year?”; “Have you engaged in leisure activities in the past year?”; “Have you participated in educational (learning) activities in the past year?” Social activities were related to clubs, friendships, political or social organizations, and volunteer activities. The following yes/no questions were used to assess participation in social activities: “Have you participated in clubs, social organizations, or political and social organizations in the past year?”; “Have you participated in volunteer activities in the past year?”

The dependent variables measured participants’ health status and included subjective health, cognitive health, and chronic diseases. Subjective health was assessed using the following question: “What do you think about your usual health condition?” The possible responses to this question were as follows: (1) very healthy; (2) healthy; (3) just so; (4) poor health; and (5) very poor health. The Mini Mental State Examination for Dementia Screening (MMSE-DS) was used to evaluate cognitive health; it consists of 19 items, including 10 on time and place, 1 on concentration, 2 on memory, 3 on language ability, 1 on composition ability, and 2 on judgment ability. Responses were scored and judged based on a total score of 30 points. Finally, the participants also responded to questions regarding the total number of chronic diseases diagnosed by a doctor.

### 2.3. Research Methods

We conducted frequency analysis to evaluate participants’ characteristics. Thereafter, regression analyses were performed to examine the effects of community environment, leisure, and social activities. SPSS WIN 24.0 was used for frequency and regression analyses. The mediating effects were analyzed using hierarchical regression analysis and bootstrapping for Hayes’ Process Macro using SPSS WIN 24.0.

## 3. Results

### 3.1. Participants’ Characteristics

Table 2 presents the participants’ characteristics. The majority of participants were female (1432; male: 944), and the number of participants in each age group was 70s (1168), 60s (613), 80s (561), and 90s (34). Education was in the order of elementary school (906), middle school (569), high school (456), uneducated (334), and college or more (111), and housing types were apartments (1138), detached houses (797), multiplex housing (377), and others (12). The most prevalent family types were older adult couples (1171) and single families (797). Many participants evaluated their own health as normal (923), good (673), or bad (635), and most considered their cognitive health to be normal (1446) or suspected dementia (507). Most participants had at least 2 (850) or 3 (629) chronic diseases. Almost an even number of participants responded that they did or did not engage in periodic physical activity: no (1197) and yes (1179).

### 3.2. Effects of Community Environment on Leisure and Social Activities among Korean Older Adults with Diabetes

Table 3 shows the result of the regression analysis to examine the effect of the community environment on participation in leisure and social activities among Korean older adults diagnosed with diabetes. Among the sub-factors of community environment, accessibility did not have a significant effect on either leisure or social activities. Satisfaction, the second sub-factor of community environment, was found to have a significant effect on both leisure (*β* = 0.105, *p* < 0.001) and social activities (*β* = 0.138, *p* < 0.001).

#### 3.2.1. Effects of Community Environment Accessibility on Leisure and Social Activities among Korean Older Adults with Diabetes

Table 4 presents the result of the regression analysis on the effect of access to the community environment on leisure and social activities. Access to convenient facilities had a significant effect on leisure (*β* = 0.063, *p* < 0.05) and social activities (*β* = 0.110, *p* < 0.001). Access to medical and health facilities was found to have a significant effect on social activities (*β* = 0.075, *p* < 0.05), whereas access to welfare facilities was found to have a significant effect on leisure activities (*β* = −0.058, *p* < 0.05).

#### 3.2.2. Effect of Community Environment Satisfaction on Leisure and Social Activities among Korean Older Adults with Diabetes

Table 5 presents the result of the regression analysis on the effect of community environmental satisfaction on leisure and social activities. Satisfaction with facilities and safety (*β* = 0.078, *p* < 0.01) and satisfaction with social interactions (*β* = 0.092, *p* < 0.01) were found to have a significant effect on social activities.

### 3.3. Effects of Community Environment on Health Status among Korean Adults with Diabetes

Table 6 shows the result of the regression analysis examining the effect of the community environment sub-factors on the health status of Korean older adults diagnosed with diabetes. Among the sub-factors of community environment, accessibility was found to have a significant effect on subjective health (*β* = −0.048, *p* < 0.05). Satisfaction was found to have a significant influence on subjective health (*β* = 0.275, *p* < 0.001), cognitive health (*β* = 0.129, *p* < 0.001), and number of chronic diseases (*β* = 0.080, *p* < 0.01).

#### 3.3.1. Effects of Community Environment Accessibility on Health Status of Korean Adults with Diabetes

Table 7 presents the result of the regression analysis examining the effect of community environment accessibility on health status. Access to convenience facilities was found to have a significant effect on subjective health (*β* = −0.082, *p* < 0.01), cognitive health (*β* = 0.144, *p* < 0.001), and number of chronic diseases (*β* = −0.137, *p* < 0.001). Access to medical and health facilities significantly affected subjective health (*β* = 0.156, *p* < 0.001), while access to welfare facilities significantly affected the number of chronic diseases (*β* = 0.120, *p* < 0.001).

#### 3.3.2. Effects of Community Environment Satisfaction on Health Status of Korean Older Adults with Diabetes

Table 8 presents the result of the regression analysis examining the effect of community environment satisfaction on health status. Satisfaction with facilities and safety had a significant effect on subjective health (*β* = 0.056, *p* < 0.05) and cognitive health (*β* = 0.058, *p* < 0.05). Meanwhile, social interaction significantly influenced subjective health (*β* = 0.218, *p* < 0.001), cognitive health (*β* = 0.101, *p* < 0.001), and number of chronic diseases (*β* = 0.114, *p* < 0.001).

### 3.4. Effects of Leisure and Social Activities on Health Status of Korean Older Adults with Diabetes

Table 9 presents the result of the regression analysis examining the effect of leisure and social activities on health status. Leisure activities had a significant effect on cognitive health (*β* = 0.042, *p* < 0.05), while social activities had a significant effect on subjective health (*β* = 0.287, *p* < 0.001), cognitive health (*β* = 0.241, *p* < 0.001), and number of chronic diseases (*β* = 0.086, *p* < 0.001).

### 3.5. Mediating Effect of Leisure and Social Activities on the Relationship between Community Environment and Health Status of Korean Older Adults with Diabetes

The hierarchical regression analysis proposed by Baron and Kenny [16] was conducted to verify the mediating effect of leisure and social activities on the relationship between community environment and the health status of Korean older adults with diabetes. Table 10 presents the results of the mediation analysis. In the regression analysis using leisure and social activities as a dependent variable, community environment had a significant effect on leisure and social activities (*β* = 0.113, *p* < 0.001). In the regression analysis in the second stage, with health status as a dependent variable, community environment was found to have a significant effect on health status (*β* = 0.171, *p* < 0.001). In the regression analysis in which all three independent variables and parameters were inputted, community environment had a significant effect on health status (*β* = 0.145, *p* < 0.001), and leisure and social activities also had a significant effect on health status (*β* = 0.225, *p* < 0.001). The effect in the third stage was partially mediated because it was less than the effect in the second stage.

**Figure 1:**
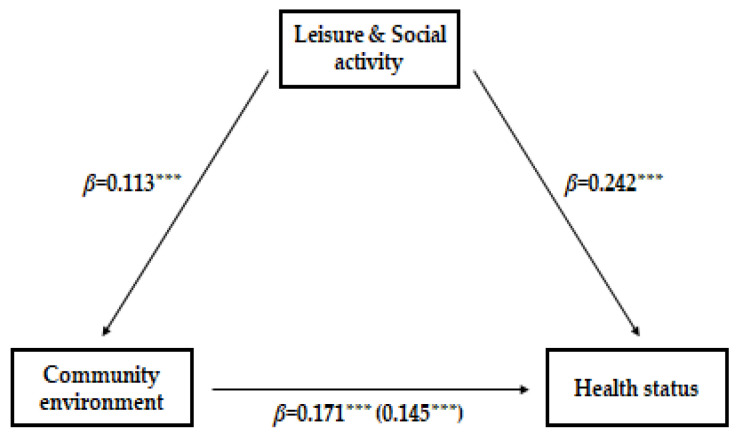
Direct and indirect effects of community environment, leisure and social activities, and health status. *** *p* < 0.001.

To verify the statistical significance of the mediating effect, bootstrapping was performed using the mediation conditional process analysis proposed by Hayes [17]; the results are shown in Table 11. The significance of the indirect effect through the bootstrapping results was determined by whether zero was included in the 95% confidence interval. The lower and upper limits of the confidence interval of the mediating effect coefficient were 0.0200 and 0.0438, respectively. As indicated by Preacher and Hayes [18], the mediating effect of leisure and social activities is statistically significant because it does not include zero in this interval. The final research model is shown in Figure 1.

## 4. Discussion

The purpose of this study was to examine the relationship between community environment, leisure and social activities, and the health status of Korean older adults with diabetes and to examine the mediating effect of leisure and social activities on the effect of community environment on health status. The implications of these results are as follows.

First, it was found that the community environment had a significant effect on participation in leisure and social activities. Specifically, access to the community environment did not have a significant effect, but satisfaction with the community environment had a significant effect on both leisure and social activities. The analysis revealed that satisfaction with facilities, safety, and social exchange opportunities significantly affected participation in social activities. This is similar to the results of previous studies [19], which showed that a positive community environment promotes participation in community life. It is also similar to the finding that older people depend more on their social relationships [7] and spend more time interacting with their neighbors than do young adults [8]. Older individuals in different environmental conditions participate in leisure and social activities based on their subjective satisfaction rather than their actual distance from the community environment. The results of this study show that community environment satisfaction is more important to older adults than access to the environment; when older adults are satisfied with the community environment, they can experience resocialization through social activities. Social activities in older age have replaced the loss of social roles due to retirement. Social lifestyles are helpful in the treatment and prevention of diseases such as diabetes [11]. This means that social activities are important for all ages, including older adults.

Therefore, as the central period of life in Korean society shifts to older age, it is necessary to provide a place for social exchange for older adults’ continuation of social activities that enable them to play a social role.

Second, community environment was found to have a significant effect on health status. Specifically, the accessibility of the community environment was found to have a significant effect on subjective health, a sub-factor of health status; moreover, satisfaction with the community environment was found to have a significant effect on all sub-factors of health status: subjective health, cognitive health, and chronic diseases. As a result of deconstructing and analyzing these effects, this study found that the better the access to convenience facilities, the worse the subjective health, the higher the cognitive health, and the lower the number of chronic diseases. Furthermore, the better the environment and accessibility related to medical and health facilities, the better the subjective health, and the better the access to facilities related to welfare, the higher the number of chronic diseases.

Based on these findings, we can observe that the environment related to medical care and health and welfare facilities affects the health of older adults with diabetes. However, accessibility to amenities closest to their daily lives had a negative effect on health, indicating that increased accessibility through quantitative increases could be a factor that worsens health. Increased accessibility can lead to decreased physical activity. In particular, the number of convenience facilities close to older adults’ daily lives is higher than that of other facilities, and the development of delivery services has had a greater effect on the decrease in physical activity. This environment has a positive effect on convenience, but it also has a negative effect in that it causes diseases such as obesity and diabetes through a reduction in physical activity [20]. Walking, the most basic method of human movement, has a great impact on health and is especially effective for diabetes [13,21]. Life can be made easier for older adults by increasing their accessibility through the quantitative and systematic increase in various facilities. However, it is necessary to provide an environment where self-health care is possible by ensuring that basic physical activity is also possible.

The higher the satisfaction with facilities and safety, the better the subjective and cognitive health, and the higher the satisfaction with social exchange, the better the subjective and cognitive health and chronic diseases. This result can be interpreted as follows: the higher the accessibility of the most frequently used convenience facilities, the lower the amount of activity and the worse the health of older adults, and the greater the opportunities for social exchange, the better the overall health status. This was similar to the results of previous studies [22] that reported that quality of life increases if one can live in harmony with the community environment. Therefore, providing an environment for health activities for older adults with confirmed diabetes will improve their lifestyle, health status, and quality of life [23]. Taken together, the importance of welfare facilities, which are the main social exchange spaces for older adults, needs to be recognized. Welfare facilities for older adults are important for social exchanges and physical activities, as they provide the space for performing social and physical activities that are lacking in everyday life. This can be interpreted as having a positive effect on subjective health, cognitive health, and chronic disease, which positively influences health in older age. The primary task facing the aging Korean society is expanding the infrastructure for older adults and enabling its systematic operation through institutional development by fostering experts in the field of physical activity for older adults.

Third, leisure and social activities had significant effects on health status. Specifically, leisure activities significantly affected cognitive health, whereas social activities significantly affected subjective health, cognitive health, and chronic diseases. This result is similar to that of a previous study in which leisure activities boosted mental health, and to that of another study [24,25], which highlighted that the presence of social activities affects physical and mental health of diabetic older adults [26,27,28]. The American Heart Association [29] recommends participation in personal and social activities for a certain period to ensure the health of older adults [30]. This means that engaging in social activities, interacting with others, and resocializing have a positive impact on the health of older adults and, subsequently, on their quality of life in older age. Abundant social interactions and active lifestyles offset harmful diseases such as diabetes [11,31]. This means that social activities are more effective than leisure activities for older adults who have experienced desocialization, and that both leisure activities and social activities are major factors affecting health for older adults suffering from diabetes in Korea. In older age, an individual’s overall life is centered on the family rather than society [6], but their need for activities and spaces that can be matched with peers of various ages is greater.

Finally, the partial mediating effect of leisure and social activities was found to be significant in the relationship between community environment and health status. This means that to maintain and promote the health of older adults diagnosed with diabetes, it is necessary to improve the community environment and increase opportunities for participation in leisure and social activities. In particular, opportunities for social exchange, which have a positive impact on subjective and cognitive health and the number of chronic diseases in community environments, should be increased. For older adults who have experienced desocialization, social activities with similar peers and communities eventually lead to healthy aging [26,32]. In particular, creating a community environment that can provide social exchange has been demonstrated to be important for improving the health of older adults diagnosed with diabetes [33], and efforts are being made to establish such an environment. However, mere quantitative increases will not be sufficient and could have a negative impact. Therefore, it is necessary to cultivate and manage professionals, such as senior administrative experts and physical activity experts, who can increase opportunities for participation by increasing social exchanges for older adults and systematically managing their mental and physical health.

## 5. Conclusions

This study investigated the effects of community environment, leisure, and social activities on the health status of older adults with diabetes, a serious disease in modern society. Data from the 2020 National Survey of Older Koreans were used. Descriptive statistics were used to assess participants’ characteristics, and regression analyses were conducted to assess the effects of community environment, leisure, and social activities. Mediating effects were tested using hierarchical regression analysis and bootstrapping. The key results are as follows: (a) Community environmental satisfaction affected participation in leisure and social activities. (b) Community accessibility had a negative effect on subjective health, while community environmental satisfaction had a positive effect on subjective health, cognitive function, and chronic diseases. (c) Leisure activities had a positive effect on cognitive health, while social activities influenced subjective health, cognitive function, and chronic diseases. (d) Analysis of the mediating effect of leisure and social activities on the relationship between the community environment and the health status of older adults with diabetes confirmed a partial mediating effect.

This study confirmed the influence of community environment, leisure, and social activities on the health of older adults with diabetes in Korea. Increasing accessibility was expected to have a positive impact on health, but it was found to have a negative impact. This suggests that quantitative increases are important, but quality improvements are a critical aspect. In order to improve the mental and physical health of older adults, mere quantitative increases in the community environment will not be sufficient. It is necessary to cultivate and manage professionals to increase opportunities for participation by increasing social exchanges and systematically managing older adults’ mental and physical health. The welfare system is being prepared for various characteristics of older adults and specific diseases. The results of this study could provide basic data for preparing and operating the welfare system and could, thus, improve the health of older adults with diabetes in Korea.

Despite its strengths, this study has some limitations. First, the survey of older adults should include a larger number of questions for older adults aged over 65 years. In this process, the reliability of the answers could be suspect. Second, the responses to the survey questions were very limited. Third, the survey of older adults was a one-time survey. In future studies, it will be necessary to conduct individual and longitudinal research to verify the survey’s effectiveness.

## Figures and Tables

**Table 1:** Description of variables.

Variable Type	Variable Name	Sub-Factors
Independent variable	Community environment	Accessibility
		Satisfaction
Mediation variable	Leisure and Social activities	Leisure activities
		Social activities
Dependent variable	Health status	Subjective health
		Cognitive health
		Chronic diseases

**Table 2:** Participants’ characteristics.

	Division	Cases	Percent (%)		Division	Cases	Percent (%)
Sex	Male	944	39.7	Subjective health	Very good	70	2.9
	Female	1432	60.3				
Age	60s	613	25.8		Good	673	28.3
	70s	1168	49.2		Normal	923	38.8
	80s	561	23.6		Bad	635	26.7
	90s	34	1.4		Very bad	75	3.2
Education	Uneducated	334	14.1	Cognitive health	Normal	1446	60.9
	Elementary school	906	38.1		Suspected dementia	507	21.3
	Middle school	569	23.9		Mild dementia	270	11.4
	High school	456	19.2		Moderate dementia	97	4.1
	College or more	111	4.7		Severe dementia	56	2.4
Housing type	Detached house	959	40.4	Chronic disease	1	258	10.9
	Apartment	1138	47.9		2	850	35.8
	Multiplex housing	267	11.2		3	629	26.5
	Other	12	0.5		4	363	15.3
Family type	Single family	797	33.5		5 or more	276	11.6
	Older adult couple household	1171	49.3	Physical activity	Yes	1179	49.6
	Cohabitation with children	377	15.9				
	Cohabitation with other family	31	1.3		No	1197	50.4

**Table 3:** Effects of community environment on leisure and social activities.

Variable	Leisure Activities		Social Activities	
	*β*	*t*	*β*	*t*
Accessibility	−0.041	−1.669	0.035	1.426
Satisfaction	0.105	4.295 ***	0.138	5.658 ***

*** *p* < 0.001.

**Table 4:** Effects of community environment accessibility on leisure and social activities.

Variable	Leisure Activities		Social Activities	
	*β*	*t*	*β*	*t*
Amenities	0.063	2.159 *	0.110	3.782 ***
Medical and health	0.023	0.710	0.075	2.369 *
Welfare	−0.058	−2.251 *	−0.045	−1.762

* *p* < 0.05, *** *p* < 0.001.

**Table 5:** Effects of community environment satisfaction on leisure and social activities.

Variable	Leisure Activity		Social Activities	
	*β*	*t*	*β*	*t*
Facility and safety	0.044	1.551	0.078	2.789 **
Social interchange	0.046	1.624	0.092	3.278 **

** *p* < 0.01.

**Table 6:** Effect of community environment on health status.

Variable	Subjective Health		Cognitive Health		Chronic Diseases	
	*β*	*t*	*β*	*t*	*β*	*t*
Accessibility	−0.048	−1.996 *	0.029	1.188	−0.007	−0.274
Satisfaction	0.275	11.525 ***	0.129	5.285 ***	0.080	3.250 **

* *p* < 0.05, ** *p* < 0.01, *** *p* < 0.001.

**Table 7:** Effects of community environment accessibility on health status.

Variable	Subjective Health		Cognitive Health		Chronic Diseases	
	*Β*	*t*	*β*	*t*	*β*	*t*
Amenities	−0.082	−2.808 **	0.144	4.938 ***	−0.137	−4.724 ***
Medical and health	0.156	4.901 ***	−0.034	−1.066	0.046	1.461
Welfare	0.041	1.574	0.017	0.649	0.120	4.634 ***

** *p* < 0.01, *** *p* < 0.001.

**Table 8:** Effects of community environment satisfaction on health status.

Variable	Subjective Health		Cognitive Health		Chronic Diseases	
	*β*	*t*	*β*	*t*	*β*	*t*
Facility and safety	0.056	2.033 *	0.058	2.049 *	−0.028	−0.988
Social interchange	0.218	7.928 ***	0.101	3.583 ***	0.114	4.011 ***

* *p* < 0.05, *** *p* < 0.001.

**Table 9:** Effects of leisure and social activities on health status.

Variable	Subjective Health		Cognitive Health		Chronic Diseases	
	*β*	*T*	*β*	*t*	*β*	*t*
Leisure activities	0.039	1.869	0.042	1.985 *	0.007	0.322
Social activities	0.287	13.759 ***	0.241	11.396 ***	0.086	3.937 ***

* *p* < 0.05, *** *p* < 0.001.

**Table 10:** Analysis of the mediating effects of leisure and social activities.

Variable		B	SE	*β*	*t*	*p*	F (p)	*p*
1	Community environment → Leisure and social activities	0.110	0.020	0.113	5.546 ***	0.000	30.763 ***	0.000
2	Community environment → Health status	0.212	0.025	0.171	8.448 ***	0.000	71.376 ***	0.000
3	Community environment /leisure and social activities → Health status	0.180	0.025	0.145	7.332 ***	0.000	102.271 ***	0.000
	Leisure and social activities → Health status	0.288	0.025	0.225	11.371 ***	0.000		

*** *p* < 0.001.

**Table 11:** Bootstrapping results of the mediating effects of leisure and social activities.

Variable	Mediating Effect Coefficient	Bootstrap Standard Error	95% Bias-Corrected CI	
			LLCI	ULCI
Leisure and social activities	0.0316	0.0061	0.0200	0.0438

CI, confidence interval; LL, lower limit; UL, upper limit.

## Data Availability

Apply for the raw data (2020 National Survey of Older Koreans) at https://data.kihasa.re.kr/kihasa/main.html (accessed on 3 May 2023).
